# In Vitro Combinatorial Activity of Direct Acting Antivirals and Monoclonal Antibodies against the Ancestral B.1 and BQ.1.1 SARS-CoV-2 Viral Variants

**DOI:** 10.3390/v16020168

**Published:** 2024-01-23

**Authors:** Lia Fiaschi, Camilla Biba, Ilenia Varasi, Niccolò Bartolini, Chiara Paletti, Federica Giammarino, Francesco Saladini, Maurizio Zazzi, Ilaria Vicenti

**Affiliations:** Department of Medical Biotechnologies, University of Siena, 53100 Siena, Italy; lia.fiaschi@unisi.it (L.F.); camilla.biba@student.unisi.it (C.B.); ilenia.varasi@student.unisi.it (I.V.); niccolo.bartolini@student.unisi.it (N.B.); c.paletti@student.unisi.it (C.P.); federica.giammari@gmail.com (F.G.); saladini6@unisi.it (F.S.); maurizio.zazzi@unisi.it (M.Z.)

**Keywords:** SARS-CoV-2, mAb, DAA, combination antiviral therapy, in vitro synergism, Omicron variant

## Abstract

Combination antiviral therapy may be helpful in the treatment of SARS-CoV-2 infection; however, no clinical trial data are available, and combined use of direct-acting antivirals (DAA) and monoclonal antibodies (mAb) has been reported only anecdotally. To assess the cooperative effects of dual drug combinations in vitro, we used a VERO E6 cell-based in vitro system with the ancestral B.1 or the highly divergent BQ.1.1 virus to test pairwise combinations of the licensed DAA, including nirmatrelvir (NRM), remdesivir (RDV) and the active metabolite of molnupiravir (EIDD-1931) as well the combination of RDV with four licensed mAbs (sotrovimab, bebtelovimab, cilgavimab, tixagevimab; tested only with the susceptible B.1 virus). According to SynergyFinder 3.0 summary and weighted scores, all the combinations had an additive effect. Within DAA/DAA combinations, paired scores with the B.1 and BQ.1.1 variants were comparable. In the post hoc analysis weighting synergy by concentrations, several cases of highly synergistic scores were detected at specific drug concentrations, both for DAA/DAA and for RDV/mAb combinations. This was supported by in vitro confirmation experiments showing a more than a linear shift of a drug-effective concentration (IC_50_) at increasing concentrations of the companion drug, although the effect was prominent with DAA/DAA combinations and minimal or null with RDV/mAb combinations. These results support the cooperative effects of dual drug combinations in vitro, which should be further investigated in animal models before introduction into the clinic.

## 1. Introduction

Severe Acute Respiratory Syndrome Coronavirus 2 (SARS-CoV-2), which causes coronavirus disease 2019 (COVID-19), has expanded worldwide since its emergence in China in December 2019, resulting in a global pandemic with more than 700 million confirmed cases and about 7 million deaths documented as of September 2023 (https://COVID19.who.int/ accessed on 20 September 2023). Massive vaccination and natural SARS-CoV-2 infections and reinfections have progressively expanded immunity to SARS-CoV-2, drastically reducing the incidence of COVID-19 and its severe outcomes in the immunocompetent population. As a result, the end to COVID-19 as a global health emergency was declared in May 2023 by the World Health Organization (https://www.who.int/europe/emergencies/situations/COVID-19 accessed on 20 September 2023). However, SARS-CoV-2 continues to circulate endemically in the global population, causing serious illness in the most fragile categories, such as elderly people comorbid and immunocompromised patients [1,2]. The persistent circulation of SARS-CoV-2 in the human population has been driven by the ability to evade the host immune response, particularly since the emergence of the Omicron variant and the plethora of its sublineages, which continue to evolve rapidly and challenge population immunity [3]. In addition, waning immunity combines with decreased vaccine acceptance to pave the way to novel epidemic waves [4]. Thus, early treatment of symptomatic patients with available antiviral medications remains crucial to protect fragile individuals and limit virus spread and evolution.

Currently, available anti-SARS-CoV-2 agents include the direct-acting antivirals (DAA) remdesivir (RDV), molnupiravir (MNP), and ritonavir-boosted nirmatrelvir (NRM) as well as anti-spike monoclonal antibodies (mAb) [5]. Unfortunately, continued virus evolution has progressively decreased or abolished mAb neutralization activity, with few cases of restored activity against the currently dominating virus lineages [6]. On the other hand, DAA has maintained their activity across multiple variants due to the high conservation of the targeted viral enzymes [7]. However, current DAA may be unable to fully prevent severe disease progression, and their use is limited by a number of drug–drug interactions (NRM), inconvenient administration (RDV), or mutagenicity concerns (MNP) [8]. Indeed, the development of novel drugs or different therapeutic approaches remains a key priority [9]. While waiting for novel agents, one option recently gaining interest is to combine available drugs into a drug cocktail based on the long-standing success of combination therapy in HIV and HCV infection [10]. The key advantage of this approach is that each drug used in the combination can act at a different step in the virus replication cycle, increasing the effectiveness of the treatment while reducing the emergence of drug-resistant viral variants. Interestingly, if the drugs interact synergistically, the active dose can be reduced with respect to monotherapy, potentially lowering toxicity. Although no combination therapy is approved for the treatment of COVID-19, some studies have recently evaluated the combined effect of licensed or candidate anti-SARS-CoV-2 compounds in vitro [11,12,13,14] and in animal models [15,16] and anecdotal cases of combined administration of two anti-SARS-CoV-2 treatments in clinical practice have been reported [17,18,19,20].

In this work, we evaluated the in vitro interactions between approved anti-SARS-CoV-2 compounds in a live virus cell-based assay using the prototype SARS-CoV-2 B.1 virus and a more recent omicron lineage (BQ.1.1). We included all the three DAA (RDV, MNP, NRM) plus the four mAb which maintained activity during the initial evolution of the Omicron lineage.

## 2. Materials and Methods

### 2.1. Cells and Viral Stocks

The VERO E6 monkey cell line (ATCC^®^ CRL-1586) was used to propagate and titrate the viral stocks and to determine the antiviral activity of the antiviral compounds, alone or in combination. VERO E6 cells were propagated in DMEM High Glucose medium (Euroclone, Pero, Italy) supplemented with 10% Fetal Bovine Serum (FBS) and 1% Streptomycin/Penicillin (PS) (Euroclone, Pero, Italy) in a humidified incubator at 37 °C with 5% CO_2_. The same medium with a lower FBS concentration (1%) was used for viral infection experiments. For all DAA activity experiments, VERO E6 cells were pre-treated with 0.5 µM CP-100356 hydrochloride inhibitor to reduce the efflux activity of P-glycoprotein, which is overexpressed by this cell line [21,22].

The SARS-CoV-2 stocks used for the experiments included the wild type B.1 (GISAID code EPI_ISL_2472896) and the Omicron BQ.1.1 (EPI_ISL_17257516) variant, kindly provided by the Department of Biomedical and Clinical Sciences Luigi Sacco, University of Milan, Italy. Viral stocks were expanded in VERO E6 cells and titrated as previously described [23].

### 2.2. Drugs and Cytotoxicity Assay

The P-glycoprotein inhibitor CP-100356 (cat. HY-108347) and the DAA, including RDV (cat. HY-104077), NRM (cat. HY-138687) and EIDD-1931 (the active metabolite of MNP; cat. HY-125033), were supplied by MedChemExpress (Monmouth Junction, NJ, USA) as powder and dissolved in 100% dimethyl sulfoxide (DMSO). Biosimilar mAb, including bebtelovimab (BEB; ref. PXTA1750-100), sotrovimab (SOT; ref. PXTA1637-100), tixagevimab (TIX; ref. PXTA1032-100) and cilgavimab (CIL; ref. PXTA1033-100) were supplied by ProteoGenix SAS (Schiltigheim, France) as powder and dissolved in phosphate-buffered saline (PBS).

The cytotoxicity of all investigated compounds was assayed in VERO E6 cells as previously described [22]. Briefly, cell viability was calculated using the CellTiter Glo 2.0 Luminescent Cell Viability Assay (Promega, Madison, WI, USA), and luminescence values were measured using the GloMax^®^ Discover Multimode Microplate Reader (Promega, Madison, WI, USA) and elaborated with the GraphPad PRISM software version 9.0 (La Jolla, CA, USA). The half-maximal cytotoxic concentration (CC_50_) and the compound concentration allowing 90% cell viability (CC_10_) were calculated using a non-linear regression analysis of the dose–response curves and the ECanything GraphPad function. The CC_10_ was used for each compound as the starting concentration in the antiviral activity assay.

### 2.3. Antiviral Activity Assay

To determine the antiviral activity of each compound against the B.1 and BQ.1.1 SARS-CoV-2 variants, a direct yield reduction assay, based on the infection of cells in the presence of serial drug dilutions, was performed as previously described [22]. The day before the infection, 6000 VERO E6 cells per well were pre-seeded on 96-well adhesion plates to ensure 70% confluence on the day of infection. Cells were treated with 4-fold decreasing concentrations of each antiviral and after 1 h incubation at 37 °C with 5% CO_2_, the cells were infected at 0.005 multiplicity of infection (MOI). To test mAb activity, the viral stocks were pre-incubated at MOI 0.005 with mAb dilutions for 1 h at 37 °C and then the virus/mAb mixture was transferred onto semiconfluent pre-seeded VERO E6 cells. In both cases, after an additional 72 h incubation, cell viability was measured with CellTiter Glo 2.0. The half-maximal inhibitory concentration (IC_50_) was calculated using a non-linear regression analysis of the dose–response curves generated with GraphPad PRISM. Infected and uninfected cells without drugs were used to calculate the 0% and 100% cell viability, respectively, and used to normalize the results.

The different DAA combinations (NRM/EIDD-1931, NRM/RDV, RDV/EIDD-1931) were evaluated against the B.1 and BQ.1.1 variants. In addition, RDV only was tested in combination with the four mAb (RDV/SOT, RDV/BEB, RDV/CIL, RDV/TIX) based on convenience in the perspectives of clinical use since both RDV and mAb must be administered parenterally. These combinations could only be tested against the B.1 prototype virus because none of the mAb considered was active against the BQ.1.1 variant. The protocol previously described to determine the antiviral activity of individual compounds was adapted to the dual compound combinations. To generate the dual combinations, six 4-fold serial dilutions of the first compound were mixed with six 4-fold serial dilutions of the second compound in 96-well plates to obtain 36 different combos in a two-dimensional matrix. Briefly, for the DAA combos, the drug combination matrix was incubated for 1 h at 37 °C on pre-seeded semiconfluent VERO E6 cells, and the cultures were then infected with B.1 and BQ.1.1 viral stock at 0.005 MOI. For the mAb/antiviral combos, the B.1 viral stock was pre-incubated for 1 h at 37 °C with mAb dilutions, and then the virus/mAb mixture was transferred at 0.005 MOI onto VERO-E6 cells pretreated with serial DAA dilutions. For both conditions, infected cells were incubated for an additional 72 h at 37 °C with 5% CO_2_, and cell viability was calculated with the Cell Titer-Glo 2.0 protocol as described above. Each plate also included the dose–response curves for each individual compound, a mock infection control, and an infection control without antivirals to normalize the results. Two independent experiments were performed for each combination.

### 2.4. Dose-Effect Relationships with Compound Combinations

Antiviral activity data derived from compound combinations were analyzed using the online SynergyFinder 3.0 tool (https://synergyfinder.fimm.fi/ accessed on 12 October 2023). This version of the software was recently implemented with post-analysis options enabling better exploration and interpretation of the dose-effect relationships between antiviral agents at any tested concentration [24]. The default Zero interaction potency (ZIP) model was used to determine the type of drug–drug interaction by comparing the change in the potency of the dose–response curves between individual drugs and their combinations. The ZIP model combines the advantages of both the Loewe additivity and the Bliss independence models, aiming at a systematic assessment of various types of drug interaction patterns that may arise in a high-throughput drug combination screening [25]. Briefly, the Loewe additivity model quantifies the excess over the expected response as if the two drugs were the same drug, while the Bliss model computes the drug–drug interaction effect with respect to a multiplicative effect between two drugs acting independently from each other. For each dual compound combination, the results were expressed as a single summary Synergy Score (SS), which is averaged over all the dose combination measurements and is interpreted as the percent change in response due to drug interactions with respect to the effect of the individual drugs (e.g., a synergy score of 20 indicates 20% increased activity beyond expectation). The interaction is visualized using 2D and 3D synergy maps that highlight the synergistic and antagonistic dose areas in red and green, respectively. As per the indications of the system, an antagonistic, additive, and synergistic effect of the drug combination is defined by a summary SS value below or equal to −10, from −10 to 10, and above 10, respectively [24]. In addition, SynergyFinder 3.0 implements a “Weighting synergy by concentrations” post hoc analysis, returning a weighted SS metric as a measure of synergy at lower drug dose windows where toxicity is less likely and clinical application is favored [24].

To confirm the results obtained by SynergyFinder 3.0, we selected drug concentrations that were repeatedly detected as synergistic (SS > 10) in the drug–drug matrix and directly measured the IC_50_ shift of the drug combination in experiments where Compound 1 at three fixed concentrations (IC_50_ plus two 2-fold dilutions) was mixed with six 4-fold decreasing concentrations of Compound 2. The three Compound 2 IC_50_ values obtained in the presence of the fixed concentrations of Compound 1 were compared with the IC_50_ of Compound 2 alone, and three corresponding fold shift values in Compound 2 IC_50_ were calculated as IC_50_ [Compound 2 alone]/IC_50_ [Compound 1 + Compound 2].

### 2.5. Statistical Analysis

SynergyFinder 3.0 does not perform any statistical comparison among summary or weighted SS values obtained with different compound combinations. Since the summary SS is the average of all the individual SS obtained with all the concentration pairs tested, the overall combinatorial effects of the three DAA pairs, as well as those of the three RDV/mAb groups, were compared using the Kruskal–Wallis test, followed by Mann–Whitney pairwise comparisons between groups. SS values obtained with the same DAA/DAA concentrations against the B.1 and BQ.1.1 viral variants were compared using the Wilcoxon signed rank test. Analyses were performed using IBM SPSS Statistics, version 20 (IBM Corp., Armonk, NY, USA), and all *p*-values were 2-sided.

## 3. Results

The IC_50_ of EIDD-1931, RDV and NRM, when tested alone, were 2.40 ± 0.40/1.59 ± 0.44, 0.06 ± 0.03/0.03 ± 0.01 and 0.10 ± 0.03/0.10 ± 0.01 μM against B.1/BQ.1.1, respectively. The mAb tested, namely CIL, TIX, BEB, and SOT were active only against the wild type B.1 SARS-CoV-2 variant (IC_50_ values: 0.20 ± 0.13, 0.07 ± 0.04, 0.03 ± 0.01, 0.81 ± 0.32 µg/mL, respectively). All the IC_50_, CC_50_, and CC_10_ values are reported in Table 1.

**Figure 1:**
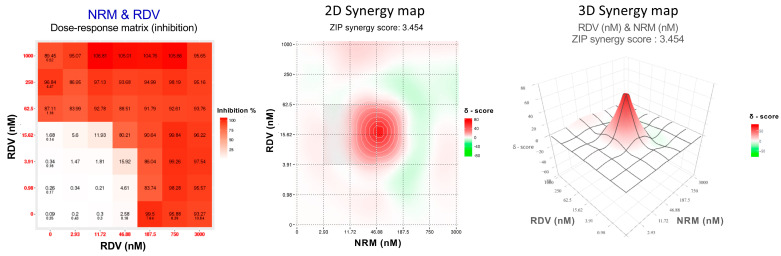
Dose–response matrix, two-dimensional (2D) and three-dimensional (3D) synergy maps generated using SynergyFinder 3.0 by applying the ZIP model on the Nirmatrelvir (NRM)/Remdesivir (RDV) combination tested against the ancestral B.1 SARS-CoV-2 strain.

The DAA combos tested against the wild type B.1 and BQ.1.1 strains were NRM/EIDD-1931, NRM/RDV, and RDV/EIDD-1931 while all mAb were tested only against the wild type B.1 strain in combination with RDV, the only injectable DAA (RDV/SOT, RDV/BEB, RDV/CIL, RDV/TIX). To evaluate DAA/DAA or RDV/mAb interactions, we analyzed the data generated from combination experiments using the SynergyFinder 3.0 software and applying the ZIP multiple synergy algorithm that combines Bliss and Loewe reference models and returns summary SS and weighted SS metrics averaging the results over all the dose combination measurements and at clinically relevant lower drug dose windows, respectively. An example of the output generated by analyzing the 36-cell matrix obtained with the NRM/RDV combo against the B.1 SARS-CoV-2 strain is shown in Figure 1. The weighted SS for all the DAA combinations indicated additivity against B.1 (2.1 ± 0.4 for NRM/EIDD-1931, 1.9 ± 1.4 for NRM/RDV and 0.9 ± 0.7 for RDV/EIDD-1931) and BQ.1.1 (0.0 ± 0.7 for NRM/EIDD-1931, 2.1 ± 2.1 for NRM/RDV and 1.7 ± 2.5 for RDV/EIDD-1931). Additivity was also observed for all RDV/mAb combos against B.1 (6.1 ± 2.9 for RDV/SOT, 4.2 ± 3.3 for RDV/BEB, 0.8 ± 0.1 for RDV/CIL and 2.6 ± 2.7 for RDV/TIX). The more comprehensive summary score metric confirmed additivity for all the combinations tested. Summary and weighted SS are indicated in Table 2, while the 2-D Synergy plots generated by each experiment are shown in Supplementary Figure S1.

Despite the overall additive effect shown by summary and weighted SS for all the combinations, there were specific drug combo concentrations below to the IC_90_ of the individual drugs, showing synergy. Table 3 shows these cases, stratified by their SS (10–29.9, 30–50, and above 50). The highest SS were obtained for several RDV/mAb concentrations, particularly with SOT, BEB, and TIX, which was also reflected by the highest overall summary and weighted SS (Table 2). Among DAA combinations, only NRM/RDV reached the highest SS stratum, but only at one concentration, while hits at lower synergistic strata were detected for NRM/EIDD-1931, RDV/EIDD-1931, and again NRM/RDV. The number of hits showing synergy for NRM/EIDD-1931, NRM/RDV, and RDV/EIDD-1931 was 5, 6, and 2, respectively. When comparing SS values obtained with all the DAA/DAA and DAA/mAb combinations and the B.1 virus, the Kruskal–Wallis test revealed significant differences (*p* < 0.001), and post hoc pairwise analysis detected significantly higher synergy for RDV/SOT compared with the other RDV/mAb combinations (RDV/BEB, *p* = 0.006; RDV/CIL, *p* < 0.001; RDV/TIX, *p* = 0.006) and with two DAA/DAA combinations (NRM/EIDD-1931, *p* < 0.001; NRM/RDV, *p* = 0.010; RDV/EIDD-1931, *p* = 0.004). However, weighted SS values indicating synergistic effects often were outliers in the overall distribution of data points for most dual combinations (Supplementary Figure S2). SS values remained significantly higher for the RDV/SOT combo when comparing only the four DAA/mAb combinations (*p* < 0.001 compared with RDV/CIL and *p* = 0.002 compared with RDV/BEB and RDV/TIX). Within the DAA/DAA combination subset, a comparable number of cases showing synergy was detected with the B.1 and BQ.1.1 viruses (8 each), and there was no statistically significant difference in the SS values when comparing B.1 to BQ.1.1 at paired DAA/DAA concentrations (*p* = 0.153).

**Figure 2:**
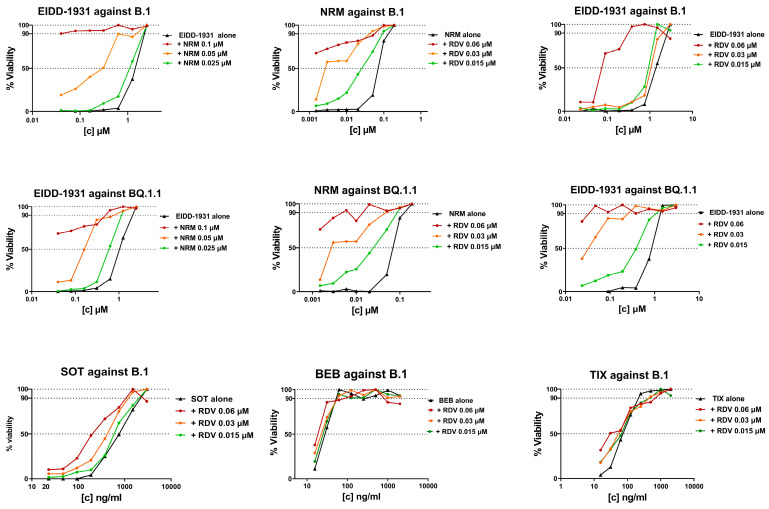
IC_50_ synergistic potency shift experiments in B.1 or BQ.1.1 virus infected VERO E6 cells treated with three fixed drug concentrations of Compound 1 (as indicated in the graph title) plus scalar concentrations of Compound 2 (as indicated in the legend). The three concentrations of Compound 1 were chosen as the IC_50_ of Compound 1 alone, plus two 2-fold dilutions. The scalar concentration of Compound 2 is the same as that used in the SynergyFinder 3.0 matrix. The concentration of Compound 2 on the *x*-axis is expressed as micromolar for DAA and as ng/mL for mAb, while the *y*-axis shows the percentage of cell viability. DAA/DAA combinations were tested against the ancestral B.1 SARS-CoV-2 strain and the BQ.1.1 Omicron variant, while RDV/mAb combinations were tested only against the ancestral B.1 SARS-CoV-2 strain. Graphs were generated using GraphPad PRISM software version 9 (La Jolla, CA, USA).

As a proof of concept, the IC_50_ synergistic potency shift was measured in infected cells treated with three fixed drug concentrations of the first compound plus scalar concentrations of the second compound. As a fixed drug, we selected NRM and RDV because they repeatedly showed synergy at specific concentrations (0.05 and 0.02 μM, respectively) close to their IC_50_ (Table 3). As shown in Figure 2 and in Supplementary Table S1, the IC_50_ of NRM with the addition of RDV 0.06 μM and 0.03 μM was reduced by 33- and 8-fold against B.1 and by >140- and 14-fold against BQ.1.1, respectively. A synergistic potency shift in EIDD-1931 IC_50_ was also induced by the addition of NRM at 0.1 μM and 0.05 μM against B.1 (88- and 11-fold, respectively) and against BQ.1.1 (28- and 7-fold, respectively) and by the addition of RDV at 0.06 μM and 0.03 μM against B.1 (>26- and 26-fold, respectively) and against BQ.1.1 (>30- and 30-fold, respectively). As shown in Figure 2 and in Supplementary Table S1, lower synergistic potency shifts were observed when fixed RDV concentrations were tested in combination with mAb. Indeed, the addition of RDV 0.06 μM reduced SOT, BEB, and TIX IC_50_ by 4-, 2- and 3-fold, respectively, while the addition of RDV 0.03 μM reduced SOT and TIX IC_50_ by 2-fold and did not affect BEB IC_50_. At the lowest concentration tested (0.015 μM), RDV induced a 2-fold reduction only in SOT IC_50_. In conclusion, the results obtained in the proof-of-concept experiments suggested a synergistic interaction with the DAA/DAA combinations; however, a cooperative effect between RDV and mAb was measurable only for RDV/SOT.

## 4. Discussion

While antiviral combination therapy has met with extraordinary success in the treatment of chronic infections, including HIV and HCV, expanding the concept to acute infections such as influenza and COVID-19 has just started to be considered [26,27]. A strong argument for combining drugs with different mechanisms of action is the need to limit the emergence of drug resistance, which is clearly a major issue with persistent viruses. However, there have been reports of rapid selection of drug resistance also with exposure of SARS-CoV-2 to mAb or DAA, particularly in immunocompromised subjects requiring prolonged treatment [28,29,30,31]. Combination therapy with SARS-CoV-2 has been so far limited to mAb cocktails, including casirivimab/imdevimab, bamlanivimab/etesevimab, and CIL/TIX. However, these combinations target the same virus replication step.

Although combined administration of two anti-SARS-CoV-2 treatments in the clinical setting has been anecdotally reported [17,18,19,20], it is currently unlikely that clinical trials comparing drug combinations to single-drug anti-SARS-CoV-2 therapy are designed. Thus, in vitro assessment of the potential for cooperative effects with multiple drugs plays a key role at this time. Of note, privileging in vitro over difficult-to-obtain in vivo data is not novel in the SARS-CoV-2 treatment arena, where authorization of mAb has been often revoked based on in vitro inefficacy data, rather than on clinical trials, with newly emerging viral variants [32]. Assessment of the dose-effect relationship with drug combinations involves measuring inhibition of viral replication in a standardized virus-cell system where different concentrations of the drugs of interest are combined. Synergistic interactions are formally defined by activity scores, which are higher than the sum of the activities of the individual drugs. Several tools are available online which are based on different mathematical models to define combinatorial effects as synergy, additivity, or antagonism (e.g., MacSynergy II™, CompuSyn, Combenefit, SynergyFinder) [33]. The available tools range from simple spreadsheet-based systems to software packages assisted by a graphical user interface and based on machine learning algorithms [34]. Since there is no consensus on how to define synergy [35], it is important to include post hoc analysis and discover the specific drug concentrations resulting in unequivocal synergy. Indeed, by using the latest release of the popular SynergyFinder, we detected highly scored synergistic potency shifts at specific concentrations of the drug combinations tested, although summary weighted SS indicated additive effects only, and, notably, we could largely support this indication using confirmatory laboratory tests.

Our results can be compared with a few published data of this kind. The NRM/EIDD-1931 combo was scored as synergistic in two previous studies, one using SynergyFinder 2.0 with the HSA algorithm to process data from a VERO E6 cell line-based system with the B.1 virus [11], the other using SynergyFinder 3.0 with the Bliss algorithm, Calu-3 cells and the Delta virus [13]. In addition, NRM and MNP were shown to increase the survival rate by >2-fold in the K18-hACE2 transgenic mouse model compared with either drug alone [16]. Another study reported an additive effect of the RDV/EIDD-1931 combination by applying the original SynergyFinder version with the ZIP algorithm in A549-ACE2-TMPRSS2 cells infected with the Omicron BA.5 virus [15]. Thus, previous reports and our study consistently show a cooperative interaction with combinations of licensed DAA. The difference between additive and synergistic effects is not straightforward to define due to the diversity of methods employed and the absence of a gold standard assay. We used the most updated version of SynergyFinder, which, in addition to the summary SS metric, also returns a weighted SS computed at lower drug concentrations and allows post hoc analysis at specific drug concentrations. Such analysis indeed revealed highly synergistic interactions, which may have driven an overall synergistic result with earlier SynergyFinder versions [11]. Synergistic hits were scored for all the three DAA/DAA combinations and for RDV with three of the four mAb considered. Indeed, our work is the first to comprehensively test all three licensed DAA and perform a first-time analysis of RDV plus mAb. Other strengths of our experimental design include the use of the CP-100356 hydrochloride drug efflux inhibitor to better mimic in vivo DAA activity and the assessment of the DAA combinatorial effects against two highly divergent viruses such as the ancestral B.1 and the later emerging Omicron BQ.1.1 variant. Comparable effects were shown with the two viral strains, as expected from the known variant-independent activity of SARS-CoV-2 polymerase and protease inhibitors [7]. A further step in this work should be updating the viral variants with those currently circulating as well as testing whether the synergistic effects measured translate into a different barrier to in vitro resistance selection.

Finally, we corroborated SynergyFinder 3.0 data with in vitro experiments to measure the IC_50_ shift for representative drug combinations where three 2-fold dilutions of one compound, downward from its IC_50_, were used together with a complete dilution series of a second compound. By selecting cases with repeated synergistic scores at concentrations close to the IC_50_, we showed that a 2-fold increase in the concentration of the first compound decreased the IC_50_ of the second compound by much more than 2-fold, supporting the synergistic effect indicated using SynergyFinder 3.0. Of note, while this was evident with DAA combinations, the effect was smaller or null with RDV/mAb combinations, advising for further studies to better define the interaction between these drugs acting at extracellular and intracellular levels. This highlights the need to support data obtained from SynergyFinder or similar tools with in vitro confirmation experiments, particularly in light of the multiplicity and frequent updates of the algorithms. In principle, any algorithm estimating combinatorial effects among drugs should be used as a starting point for further analysis and not be conclusive on its own. When a clinical trial is not feasible, advanced in vitro systems should be used, including suitable 3D lung tissue systems [36]. Most importantly, in vivo experiments should be designed in a suitable animal model before moving forward to a wide introduction of combination therapy into the clinic. SARS-CoV-2 animal models have been progressively improved [37] and should now be an integral part of the lessons learned from the COVID-19 pandemic [9].

## Figures and Tables

**Table 1:** Cytotoxicity and anti-SARS-CoV-2 activity of nirmatrelvir (NRM), EIDD-1931 (the active form of molnupiravir), remdesivir (RDV), sotrovimab (SOT), bebtelovimab (BEB), cilgavimab (CIL) and tixagevimab (TIX) in VERO-E6 cells. The antivirals were tested against the Wild Type B.1 strain and the BQ.1.1 Omicron variant. CC_50_: half-maximal toxic drug concentration; CC_10_: drug concentration causing 10% cells cytotoxicity; IC_50_: half-maximal inhibitor drug concentration; IC_90_: drug concentration inhibiting 90% of viral replication; SD: Standard Deviation; NA: Not Active.

	CC_50_ µM Mean ± SD	CC_10_ µM Mean ± SD	IC_50_ µM vs. B.1 Mean ± SD	IC_90_ µM vs. B.1 Mean ± SD	IC_50_ µM vs. BQ.1.1 Mean ± SD	IC_90_ µM vs. BQ.1.1 Mean ± SD
NRM	40.7 ± 4.0	2.9 ± 0.2	0.1 ± 0.03	0.2 ± 0.1	0.1 ± 0.01	0.2 ± 0.01
EIDD-1931	43.3 ± 6.0	3.1 ± 0.4	2.4 ± 0.4	2.5 ± 0.1	1.6 ± 0.4	1.8 ± 0.7
RDV	17.2 ± 0.2	4.2 ± 0.6	0.06 ± 0.03	0.2 ± 0.05	0.03 ± 0.01	0.1 ± 0.01
	**CC_50_ ng/mL Mean ± SD**	**CC_10_ ng/mL Mean ± SD**	**IC_50_ ng/mL vs. B.1 Mean ± SD**	**IC_90_ ng/mL vs. B.1 Mean ± SD**	**IC_50_ µM vs. BQ.1.1 Mean ± SD**	**IC_90_ µM vs. BQ.1.1 Mean ± SD**
SOT	>2400	>2400	813 ± 324	1718 ± 594	NA	NA
BEB	>60	>60	33 ± 7	89 ± 9	NA	NA
CIL	>360	>360	204 ±1354	1385 ± 862	NA	NA
TIX	>360	>360	68 ± 41	305 ± 71	NA	NA

**Table 2:** Summary synergy scores (SS) and weighted SS of all the tested antiviral combinations generated using the Synergy Finder 3.0 online software (https://synergyfinder.fimm.fi/ accessed on 12 October 2023). The antiviral pairwise combinations of nirmatrelvir (NRM), EIDD-1931 (the active form of molnupiravir), and remdesivir (RDV) were tested against the ancestral B.1 and the Omicron BQ.1.1 SARS-CoV-2 variant. The combinations of RDV with each of the four monoclonal antibodies (mAb), including sotrovimab (SOT), bebtelovimab (BEB), cilgavimab (CIL), and tixagevimab (TIX), were tested only against the ancestral B.1 SARS-CoV-2 variant. NP: Not performed.

	Summary SS vs. B.1 Mean ± SD	Weighted SS vs. B.1 Mean ± SD	Summary SS vs. BQ.1.1 Mean ± SD	Weighted SS vs. BQ.1.1 Mean ± SD
NRM/EIDD-1931	−3.1 ± 3.1	2.1 ± 0.4	−2.7 ± 1.3	0.0 ± 0.7
RDV/EIDD-1931	0.4 ± 6.2	0.9 ± 0.7	2.6 ± 6.5	1.7 ± 2.5
NRM/RDV	2.0 ± 2.0	1.9 ± 1.4	−0.2 ± 3.7	2.1 ± 2.1
RDV/SOT	8.8 ± 10.3	6.1 ± 2.9	NP	NP
RDV/BEB	5.7 ± 2.6	4.2 ± 3.3	NP	NP
RDV/TIX	0.6 ± 6.4	2.6 ± 2.7	NP	NP
RDV/CIL	−2.0 ± 0.5	0.8 ± 0.1	NP	NP

**Table 3:** Concentrations of pairwise drug combinations below each drug IC_90_ and showing synergism in the SynergyFinder 3.0 matrix, stratified by synergy score values (10–29.9, 30–50, and above 50).

Synergy Score Stratum	Drug Combination	Concentrations ^a^	Viral Variant
>50	RDV/NRM	0.02/0.05	B.1 and BQ1.1
	RDV/SOT	0.04/8.8	B.1
		0.04/35.2	
		0.04/140.6	
	RDV/BEB	0.04/0.6	B.1
		0.04/2.2	
		0.04/8.9	
	RDV/TIX	0.04/2.2	B.1
		0.04/8.8	
30–50	NRM/EIDD-1931	0.05/0.75	B.1
		0.05/1.50	B.1 and BQ.1.1
	RDV/SOT	0.01/562.5	B.1
		0.04/562.5	B.1
	RDV/TIX	0.04/35	B.1
10–29.9	NRM/EIDD-1931	0.003/0.07	B.1
		0.01/0.08	B.1
		0.05/0.09	B.1
	RDV/EIDD-1931	0.02/1.5	B.1 and BQ.1.1
		0.03/0.75	BQ.1.1
	RDV/NRM	0.02/0.05	B.1
		0.004/0.05	B.1 and BQ.1.1
		0.006/0.05	BQ.1.1
		0.02/0.03	BQ.1.1
		0.02/0.01	BQ.1.1
	RDV/BEB	0.01/8.9	B.1
	RDV/TIX	0.01/35	B.1

^a^ Concentrations are expressed as µM for DAA and ng/mL for mAb.

## Data Availability

Data are contained within the article and Supplementary Materials. All data not included are available on request from the corresponding author.

## Supplementary Materials

The following supporting information can be downloaded at: https://www.mdpi.com/article/10.3390/v16020168/s1, Table S1: IC_50_ synergistic potency shift measured in infected VERO E6 cells; Figure S1: Bi-dimensional (2D) synergy plots of antivirals against the two SARS-CoV-2 strains tested, generated using Synergy Finder 3.0 (https://synergyfinder.fimm.fi/ accessed on 12 October 2023); Figure S2: Overall combinatorial effects of the three DAA pairs as well as those of the three RDV/mAb groups compared by the Kruskal–Wallis test followed by Mann–Whitney pairwise comparisons between groups.
