# Supplementary material for: In Vitro Combinatorial Activity of Direct Acting Antivirals and Monoclonal Antibodies against the Ancestral B.1 and BQ.1.1 SARS-CoV-2 Viral Variants
Source: Viruses. 2024 Jan 23;16(2):168. doi: 10.3390/v16020168 (PMC10892871; doi:10.3390/v16020168)
Supplement: Supplementary file 1 [file viruses-16-00168-s001.zip › viruses-2822467-supplementary.pdf]

Supplementary Table S1. IC_{50} synergistic potency shift was measured in infected VERO E6 cells treated with 3 fixed drug concentrations of Compound 1 plus scalar dilution of Compound 2, for each combination. Fold shift values in Compound 2 IC_{50} were calculated as: IC_{50} [Compound 2 alone] / IC50 [Compound 1 + Compound 2]. DAA were tested against wild type B.1 SARS-COV-2 strain and BQ.1.1 variant while mAb/RDV combinations only against wild type B.1 SARS-COV-2 strain.

**Against B.1**			
**EIDD-1931 IC_{50} Fold Reduction**	**NRM 0.1 µM**	**NRM 0.05 µM**	**NRM 0.025 µM**
	88	11	1
	**RDV 0.06 µM**	**RDV 0.03 µM**	**RDV 0.015 µM**
	>26	26	2
**NRM IC_{50} Fold Reduction**	**RDV 0.06 µM**	**RDV 0.03 µM**	**RDV 0.015 µM**
	33	8	1
**SOT IC_{50} Fold Reduction**	**RDV 0.06 µM**	**RDV 0.03 µM**	**RDV 0.015 µM**
	4	2	2
**BEB IC_{50} Fold Reduction**	**RDV 0.06 µM**	**RDV 0.03 µM**	**RDV 0.015 µM**
	2	1	1
**TIX IC_{50} Fold Reduction**	**RDV 0.06 µM**	**RDV 0.03 µM**	**RDV 0.015 µM**
	3	2	1
**Against BQ.1.1**			
**EIDD-1931 IC_{50} Fold Reduction**	**NRM 0.1 µM**	**NRM 0.05 µM**	**NRM 0.025 µM**
	28	7	2
	**RDV 0.06 µM**	**RDV 0.03 µM**	**RDV 0.015 µM**
	>30	30	3
**NRM IC_{50} Fold Reduction**	**RDV 0.06 µM**	**RDV 0.03 µM**	**RDV 0.015 µM**
	140	14	2

Supplementary Figure S1: Bi-dimensional (2D) synergy plots of antivirals against the two SARS-CoV-2 strains tested, generated by Synergy Finder 3.0 (https://synergyfinder.fimm.fi/) applying the ZIP model for each experiment performed. In A were reported the 2D plots of DAAs against the wild type B.1 virus, in B the 2D plots against the BQ.1.1 variant, and in C the 2D plots of RDV/mAb combinations against the wild type B.1 virus. The results of 2 independent experiments are indicated in the upper and lower row in each box (A, B and C).

A

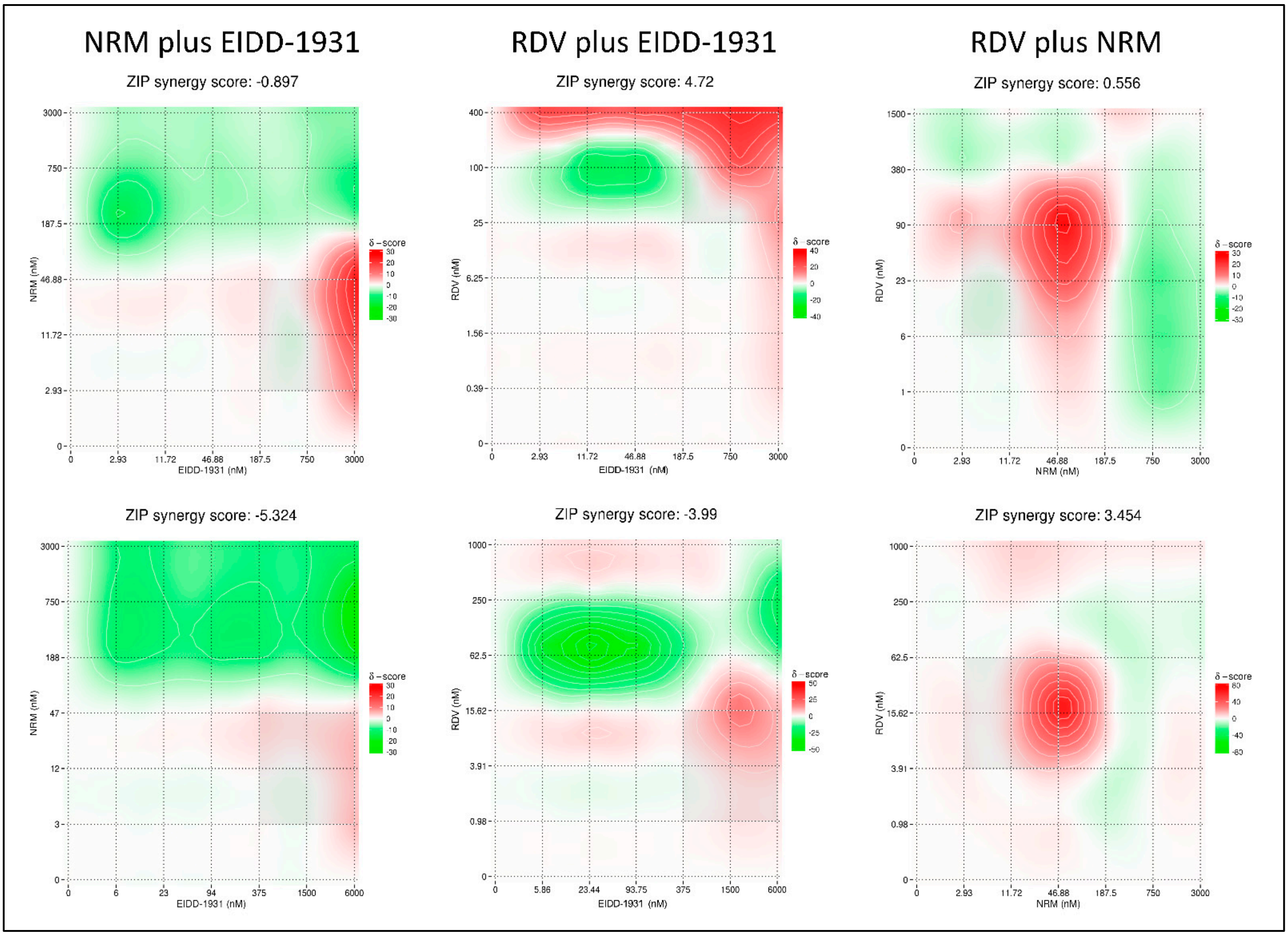

B

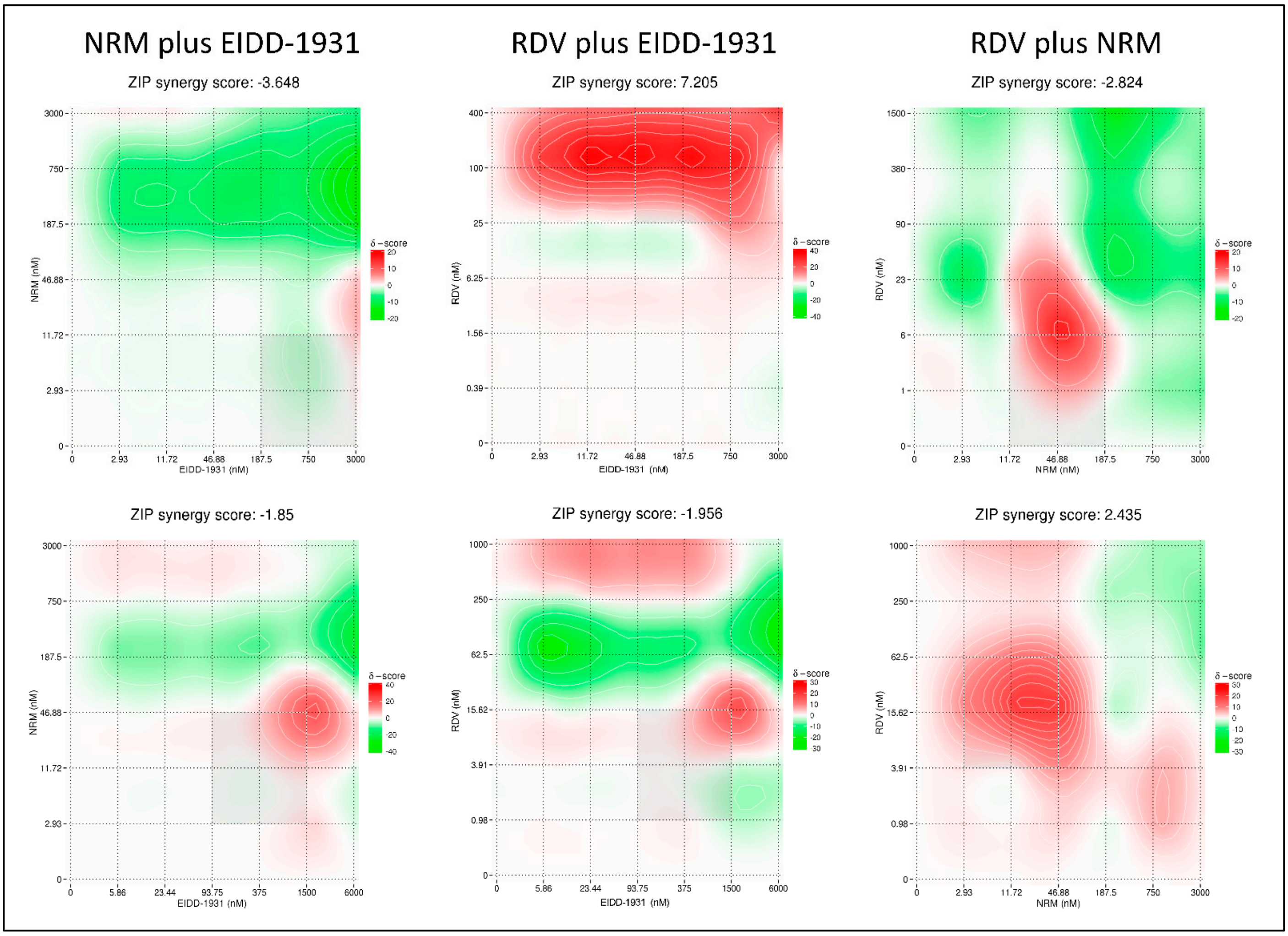

NRM plus EIDD-1931
RDV plus EIDD-1931
RDV plus NRM
ZIP synergy score: -3.648
ZIP synergy score: 7.205
ZIP synergy score: -2.824
NRM (nM)
RDV (nM)
EIDD-1931 (nM)
NRM (nM)
δ – score
ZIP synergy score: -1.85
ZIP synergy score: -1.956
ZIP synergy score: 2.435

C

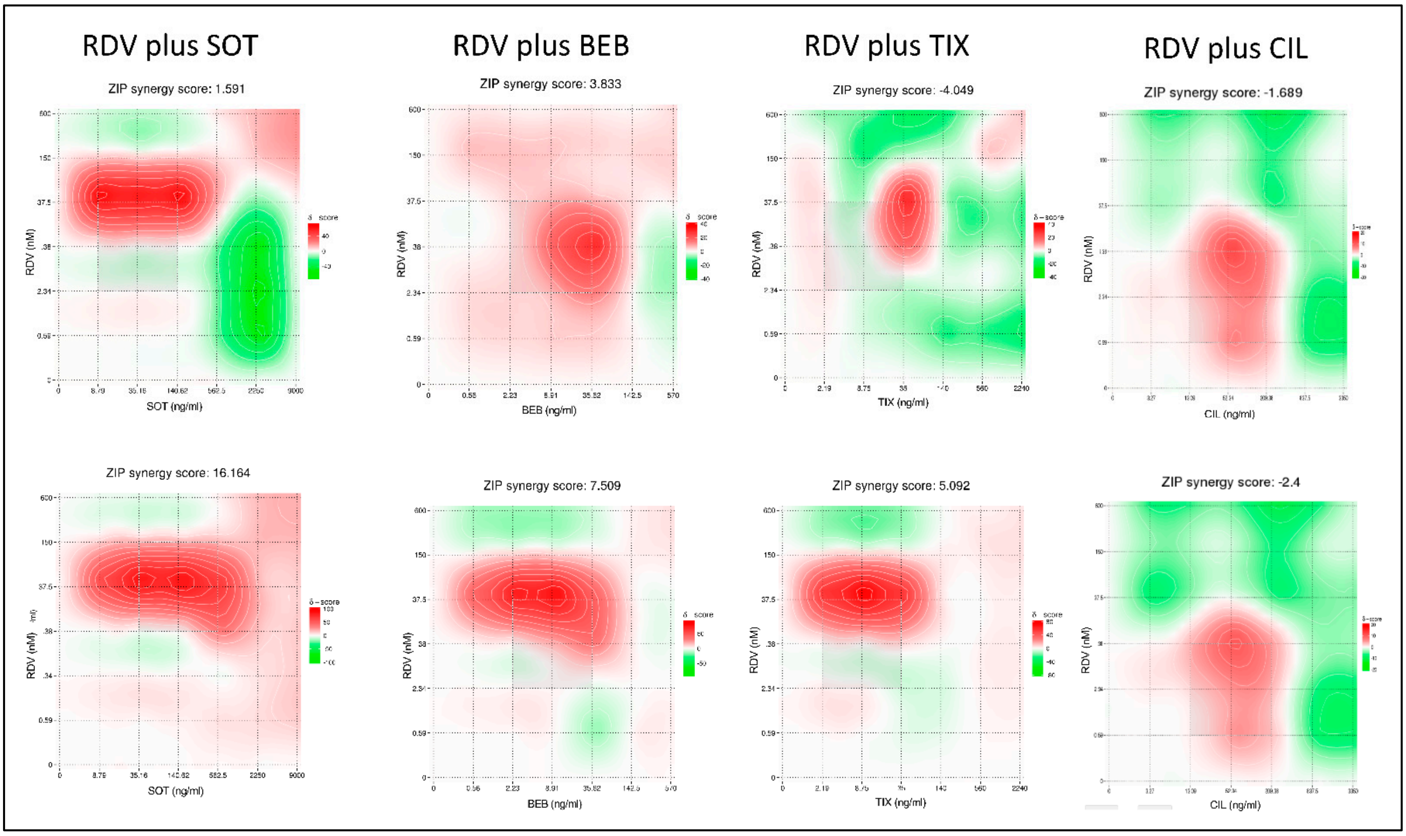

RDV plus SOT
RDV plus BEB
RDV plus TIX
RDV plus CIL
ZIP synergy score: 1.591
ZIP synergy score: 3.833
ZIP synergy score: -4.049
ZIP synergy score: -1.689
RDV (nM)
SOT (ng/ml)
BEB (ng/ml)
TIX (ng/ml)
CIL (ng/ml)
ZIP synergy score: 16.164
ZIP synergy score: 7.509
ZIP synergy score: 5.092
ZIP synergy score: -2.4

Supplementary Figure S2: Overall combinatorial effects of the three DAA pairs as well as those of the three RDV/mAb groups were compared by the Kruskal-Wallis test followed by Mann-Whitney pairwise comparisons between groups. Statistical analysis was performed by IBM SPSS Statistics, version 20 (IBM Corp., Armonk, NY, USA).

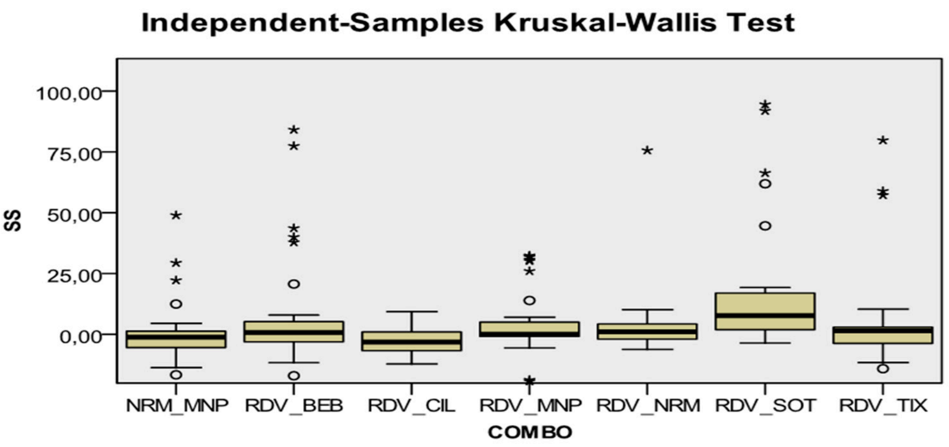

**Total N**	252
**Test Statistic**	48,797
**Degrees of Freedom**	6
**Asymptotic Sig. (2-sided test)**	,000

1. The test statistic is adjusted for ties.
